# Lichen Sclerosus of the Lip

**DOI:** 10.7759/cureus.35234

**Published:** 2023-02-20

**Authors:** Chiamaka Ohanenye, Nicholas D Brownstone, Simo Huang, Jason B Lee, Sylvia Hsu

**Affiliations:** 1 Department of Dermatology, Temple University Hospital, Philadelphia, USA; 2 Department of Dermatology and Cutaneous Biology, Thomas Jefferson University Hospital, Philadelphia, USA

**Keywords:** biopsy, lichen sclerosus et atrophicus, extragenital lichen sclerosus, oral lichen sclerosus, lichen sclerosus

## Abstract

In this case report, we outline a case of a 36-year-old woman who presented to the dermatology clinic with a history of a hypopigmented macule on her lip. After conducting hepatitis C antibody testing and a shave biopsy, the patient was diagnosed with lichen sclerosus. Because of the increased risk for squamous cell carcinoma, she underwent an anogenital exam, where no lesions were found.

## Introduction

Lichen sclerosus (LS) is a rare chronic inflammatory disorder that presents as a thin, white, or ivory-colored patch. Approximately 85% of cases of LS are seen in the anal or vulvar areas. Roughly 15-20% of patients with LS have extragenital manifestations of the condition [1]. The most common extragenital sites are the breasts, back, shoulders, neck, and thigh. Oral involvement, which includes the lips, buccal mucosa, tongue, and gingiva, has been documented but is rare. It is believed to be less destructive than LS in the genital areas [2]. Recent articles in the literature have reported fewer than 40 confirmed cases of LS exclusively found on the lips or in the oral cavity [3]. Because of the infrequent number of cases of oral LS encountered in clinical practice, the condition can often be misdiagnosed or even go undiagnosed.

## Case presentation

A 36-year-old Hispanic woman presented with a persistent lesion on her lip for two years. She reported no other associated symptoms and has no pertinent dermatologic or non-dermatologic past medical history.

**Figure 1:**
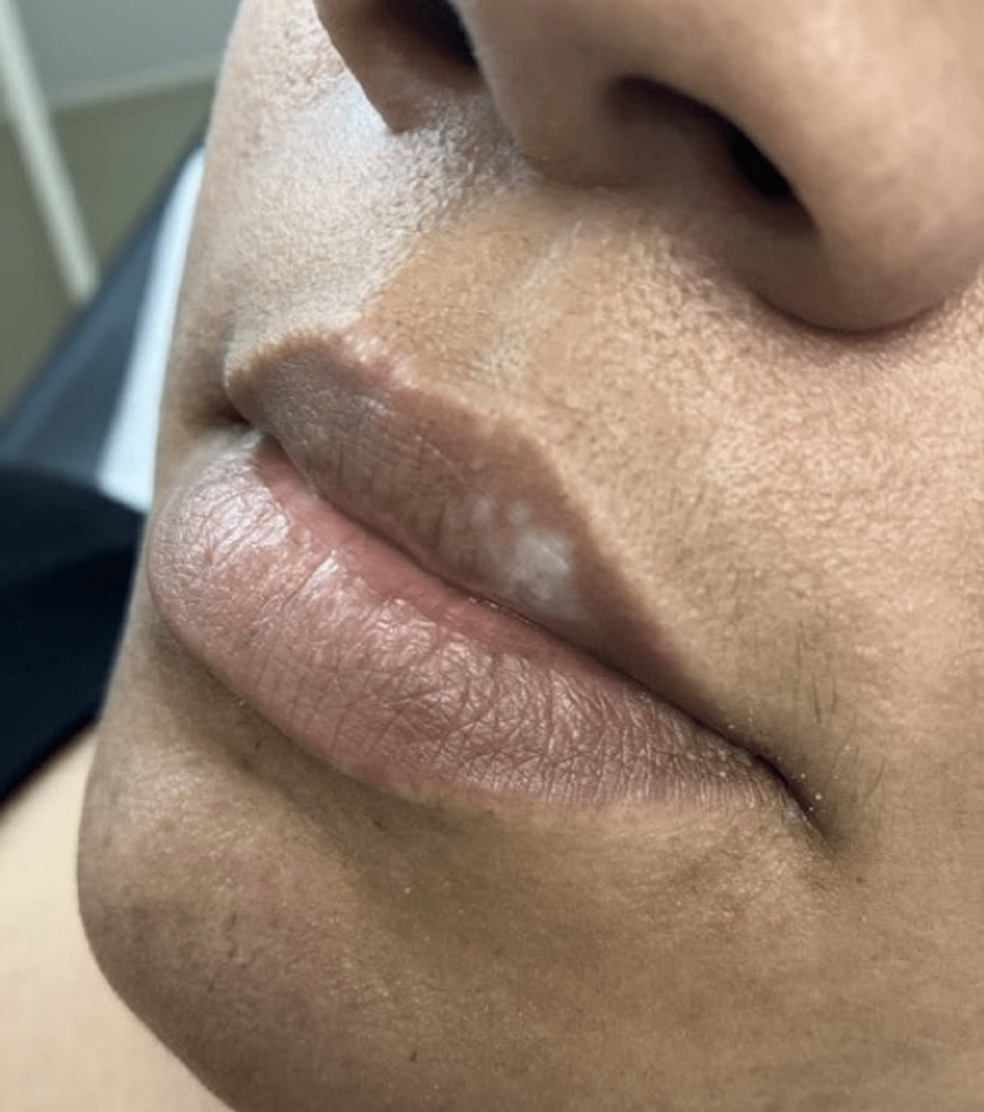
White macule on left upper lip.

Physical examination of the patient showed an 8-mm depigmented macule on the mucosal surface of the left upper lip (Figure 1).

The initial clinical diagnosis for this patient was lichen planus. We ordered a hepatitis C antibody test which was unremarkable.

**Figure 2:**
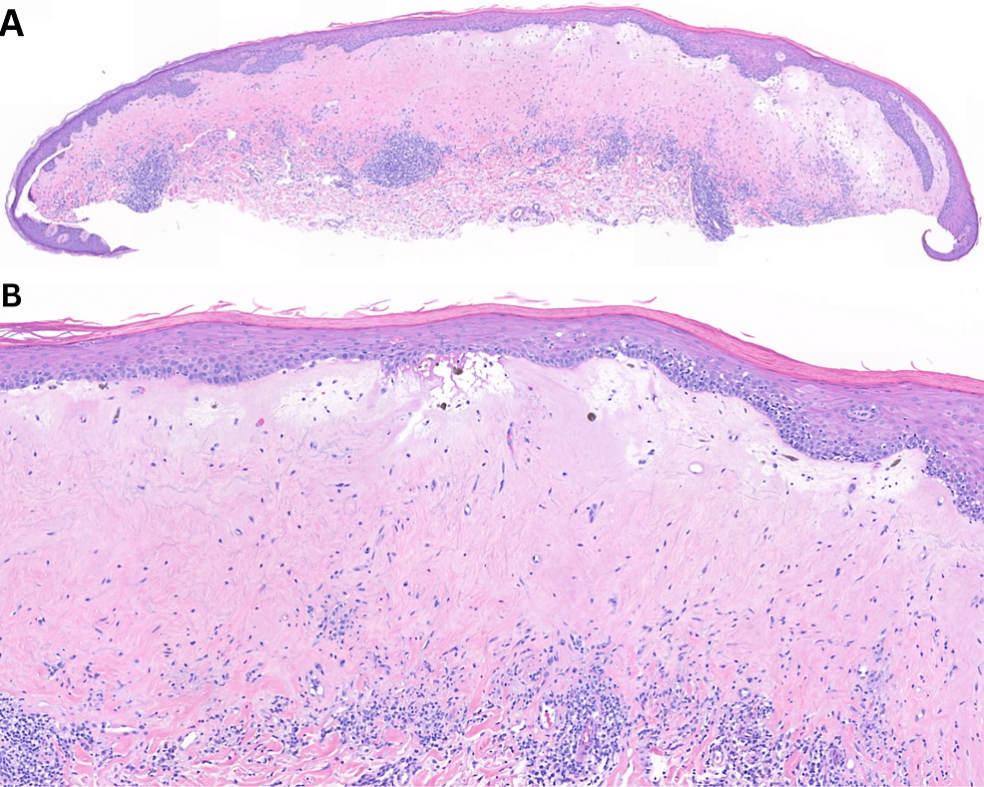
Lichen sclerosus, shave biopsy. Hematoxylin and eosin stain, (2A. 100x magnification, 2B. 200x magnification). Effacement of rete ridges and compact orthokeratosis with underlying hyalinization of the papillary dermal collagen and a band-like lymphoplasmacytic infiltrate.

A shave biopsy was performed on the left upper lip, which demonstrated effacement of rete ridges and compact orthokeratosis with underlying hyalinization of the papillary dermal collagen and a band-like lymphoplasmacytic infiltrate, consistent with LS (Figures 2A-2B). The patient returned to the clinic two weeks later, where she underwent an examination of her anogenital area. No anogenital lesions were found.

## Discussion

Diagnosing LS of the lip can be challenging because it is an uncommon location for this skin condition. It is more commonly seen in the anogenital areas. The differential diagnosis of oral LS includes vitiligo, lichen planus, lichen simplex chronicus, and leukoplakia. Distinguishing oral LS from these conditions may be difficult, which makes biopsies of any lesions critical.

Histologically, oral LS is characterized by atrophic epithelium with hyperplasia and hyperkeratosis, subepithelial hyalinization, vacuolar degeneration of the basal layer, and a lymphocytic inflammatory infiltrate with loss of elastic fibers in the upper dermis. The most important changes are seen in the superficial dermis, where edema initially becomes homogenized collagen, which is reflected in the pale staining seen in histology [1,4,5].

LS increases a patient’s risk of squamous cell carcinoma by approximately 5%. Although oral LS often appears as an isolated lesion, it may present simultaneously with anogenital LS. Because of the increased risk of cancer, if LS is seen in the mouth or another extragenital region, a careful physical exam with continuous follow-up of the affected region must be conducted.

The main goal of treatment for LS is to decrease the incidence and severity of the associated symptoms, such as pruritus and irritation, as well as to prevent inflammation in the affected areas. This is accomplished by the use of super-potent topical corticosteroids. Oral and anogenital lichenoid lesions have been shown to progress to squamous cell carcinoma in the absence of treatment [6]. Therefore, it is imperative to treat patients to prevent disease progression.

## Conclusions

In this case, we highlighted a novel and rare instance of LS found solely on the lip that was proven by histology. Although LS is most commonly seen in the anogenital area, extragenital disease should be considered if a white or ivory-colored patch or macule with scar-like atrophy is seen outside the anogenital area, such as the mucosa of the lips or oral cavity. This case contributes to the paucity of literature on LS of the peri-oral region.
